# Gene therapy and genome editing for type I glycogen storage diseases

**DOI:** 10.3389/fmmed.2023.1167091

**Published:** 2023-03-31

**Authors:** Janice Y. Chou, Brian C. Mansfield

**Affiliations:** Section on Cellular Differentiation, Division of Translational Medicine, Eunice Kennedy Shriver National Institute of Child Health and Human Development, National Institutes of Health, Bethesda, MD, United States

**Keywords:** adeno-associated virus vector, CRISPR/ Cas9 system, gene therapy, gene editing, glucose-6-phosphatase-α, glucose-6-phosphate transporter, glycogen storage disease type I

## Abstract

Type I glycogen storage diseases (GSD-I) consist of two major autosomal recessive disorders, GSD-Ia, caused by a reduction of glucose-6-phosphatase-α (G6Pase-α or G6PC) activity and GSD-Ib, caused by a reduction in the glucose-6-phosphate transporter (G6PT or SLC37A4) activity. The G6Pase-α and G6PT are functionally co-dependent. Together, the G6Pase-α/G6PT complex catalyzes the translocation of G6P from the cytoplasm into the endoplasmic reticulum lumen and its subsequent hydrolysis to glucose that is released into the blood to maintain euglycemia. Consequently, all GSD-I patients share a metabolic phenotype that includes a loss of glucose homeostasis and long-term risks of hepatocellular adenoma/carcinoma and renal disease. A rigorous dietary therapy has enabled GSD-I patients to maintain a normalized metabolic phenotype, but adherence is challenging. Moreover, dietary therapies do not address the underlying pathological processes, and long-term complications still occur in metabolically compensated patients. Animal models of GSD-Ia and GSD-Ib have delineated the disease biology and pathophysiology, and guided development of effective gene therapy strategies for both disorders. Preclinical studies of GSD-I have established that recombinant adeno-associated virus vector-mediated gene therapy for GSD-Ia and GSD-Ib are safe, and efficacious. A phase III clinical trial of rAAV-mediated gene augmentation therapy for GSD-Ia (NCT05139316) is in progress as of 2023. A phase I clinical trial of mRNA augmentation for GSD-Ia was initiated in 2022 (NCT05095727). Alternative genetic technologies for GSD-I therapies, such as gene editing, are also being examined for their potential to improve further long-term outcomes.

## 1 Introduction

Type I glycogen storage disease (GSD-I), also known as von Gierke disease, was originally considered a group of four disorders that correlated to the loss of four different protein activities: GSD-1a, the glucose-6-phosphatase-α (G6Pase-α or G6PC) activity; GSD-Ib, the glucose-6-phosphate transporter (G6PT or SLC37A4) activity; GSD-Ic, a putative phosphate transporter activity; and GSD-Id, a putative glucose transporter activity (Chou et al., 2002; Chou et al., 2010a; Chou et al., 2010b; Chou et al., 2015). Following more recent genotyping, GSD-Ic and GSD-Id cases have both been identified as harboring pathogenic *G6PT* variants, and reclassified as GSD-Ib. This is consistent with the disease biochemistry and the demonstration that G6PT is an antiporter that transports G6P from the cytoplasm into the lumen of the endoplasmic reticulum (ER), and phosphate (P_i_) in the reverse direction (Chen et al., 2008). Both GSD-Ia and GSD-Ib are autosomal recessive genetic disorders, GSD-Ia (MIM232200) representing 80% of known GSD-I cases and GSD-Ib (MIM232220) 20%. Together they have a combined incidence of 1 in 100,000 (Chou et al., 2002; Chou et al., 2010a; Chou et al., 2010b; Chou et al., 2015). Both diseases can be neonatal lethal, without intervention to stabilize blood glucose levels.

There is a third disorder, G6Pase-β (G6PC3) deficiency, also known as GSD-I-related syndrome (GSD-Irs) (Cheung et al., 2007; Boztug et al., 2009; Chou et al., 2010a; Chou et al., 2010b; Chou et al., 2015). While we will not discuss G6Pase-β deficiency in this review, it is notable that G6Pase-β is a ubiquitously expressed G6P hydrolase that can also couple with G6PT to form a G6Pase-β/G6PT complex that produces endogenous glucose in non-gluconeogenic organs (Shieh et al., 2003). Both G6Pase-α and G6Pase-β are ER-bound phosphohydrolases. Their active sites reside inside the ER lumen, and require G6PT to translocate their substrate, G6P, from the cytoplasm into the lumen (Chou et al., 2010a; Chou et al., 2010b; Chou et al., 2015). The G6Pase-α/G6PT complex is responsible for interprandial blood glucose homeostasis, while the G6Pase-β/G6PT complex is responsible for neutrophil/macrophage homeostasis and function. As a result, GSD-Ia/GSD-Ib patients have a phenotype of impaired blood glucose homeostasis, which is not present in G6Pase-β deficiency, while GSD-Ib and G6Pase-β-deficient patients have a phenotype of neutropenia and myeloid dysfunction that is not present in GSD-Ia patients.

**FIGURE 1:**
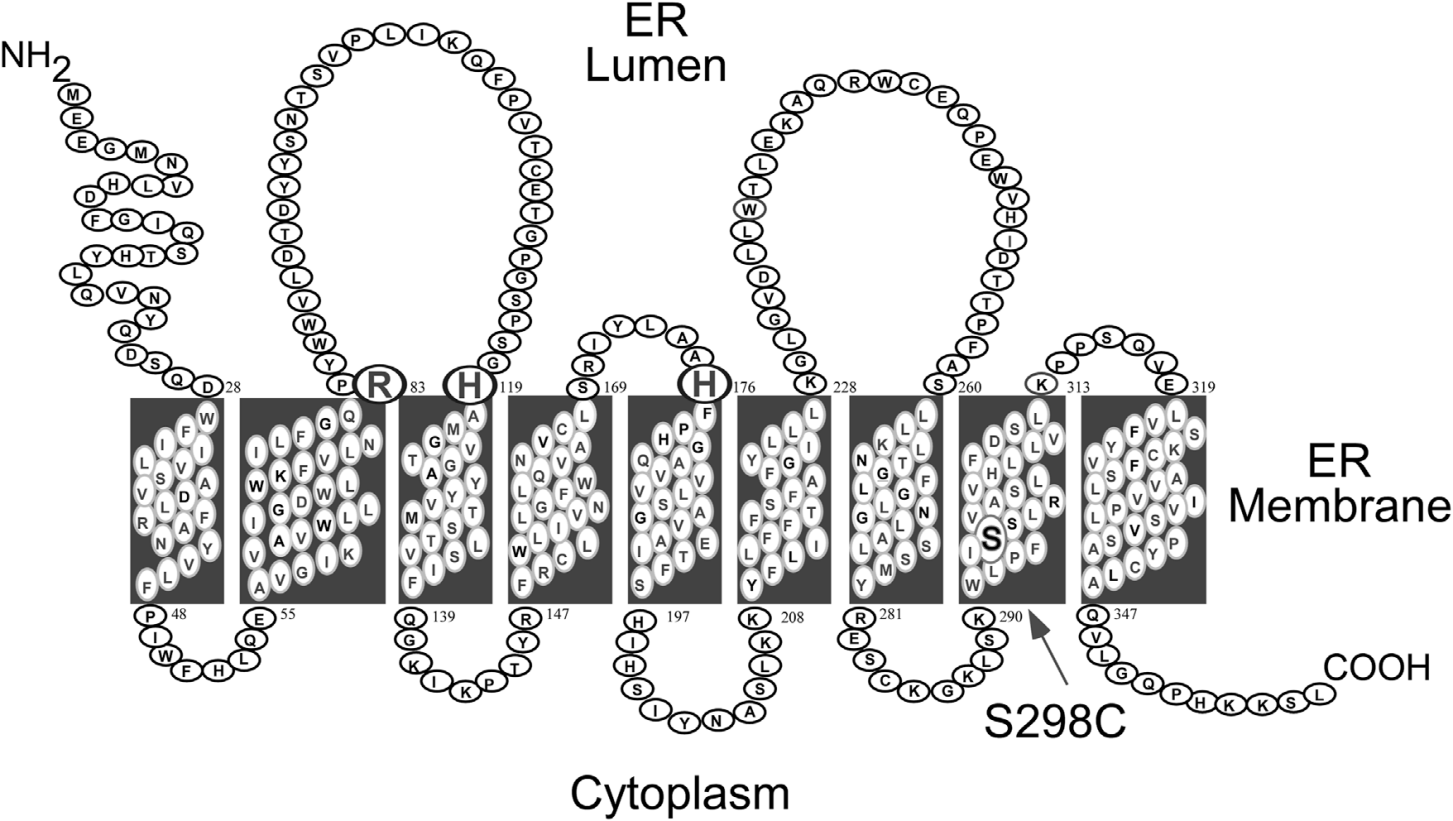
Human G6Pase-α is a 357 amino acid hydrophobic protein anchored in the ER membrane by nine helices (Pan et al., 1998). The amino acids comprising the catalytic center are highlighted by a larger font and include Arg-83, His-119, and the phosphate acceptor His-176, all situated on the luminal side of the ER (Ghosh et al., 2002). The S298C variant that increases catalytic activity (Zhang et al., 2019) is also highlighted.

GSD-Ia is caused by pathogenic variants in *G6PC,* a single copy gene on chromosome 17q21 (hg38, chr17:42,900,799-42,914,438) (Lei et al., 1993; Lei et al., 1994). It encodes the enzyme G6Pase-α, a 357 amino-acid hydrophobic transmembrane protein (Lei et al., 1993) anchored in the ER by nine helices (Pan et al., 1998) (Figure 1). G6Pase-α is expressed in a tissue-restricted manner, primarily in the gluconeogenic organs, namely, the liver, kidney cortex, and intestine (Chou et al., 2002; Chou et al., 2010a; Chou et al., 2015). The known human G6PC variants and their functional impacts have been described (Lei et al., 1995a; Lei et al., 1995b; Shieh et al., 2002).

**FIGURE 2:**
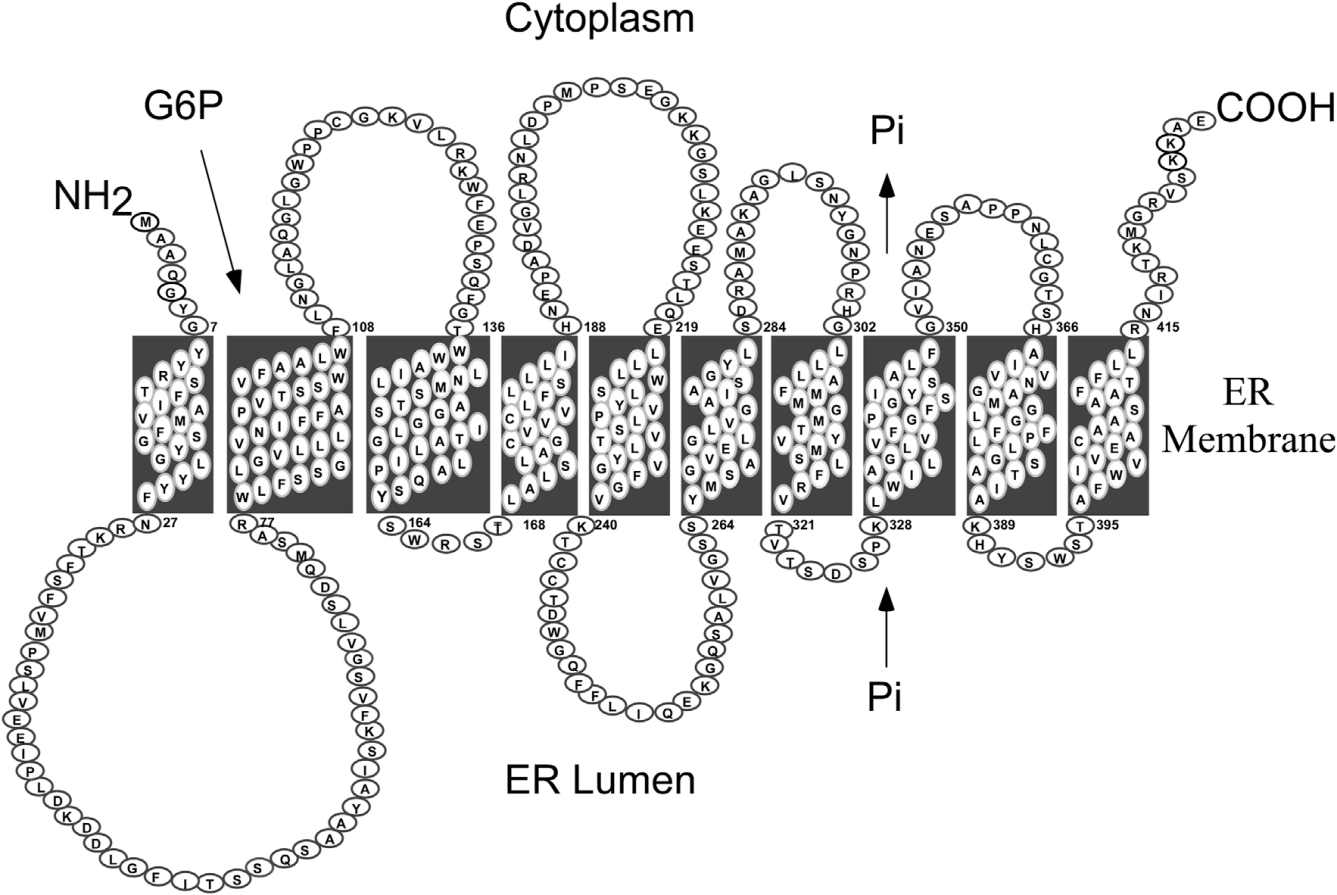
Human G6PT is a 427 amino acid hydrophobic protein anchored in the ER membrane by ten helices (Pan et al., 1999). The G6PT, also known as SLC37A4, is a P_i_-linked antiporter that transports G6P from the cytoplasm into, and P_i_ out of, the lumen of the ER (Chen et al., 2008). The G6PT is the only antiporter that can couple to G6Pase-α to form a functional G6Pase-α/G6PT complex that maintains interprandial blood glucose homeostasis (Pan et al., 2011).

GSD-Ib is caused by pathogenic variants in *SLC37A4/G6PT*, a single copy gene on chromosome 11q23 (hg38, chr11:119,024,351-119,030,906) (Annabi et al., 1998). The protein belongs to the solute carrier 37 (SLC37) family that consists of four proteins, SLC37A1, SLC37A2, SLC37A3 and G6PT/SLC37A4 (Bartoloni and Antonarakis, 2004; Chou and Mansfield, 2014). While SLC37A1, SLC37A2, and G6PT/SLC37A4 are ER-associated, P_i_-linked antiporters that can transport G6P, the G6PT/SLC37A4 is the only antiporter that can couple to G6Pase-α (Chen et al., 2008; Pan et al., 2011). The exact function of SLC37A3 is unclear. *G6PT* encodes a 427 amino-acid hydrophobic protein (Gerin et al., 1997) anchored in the ER membrane by 10 helices (Pan et al., 1999) (Figure 2). In contrast to G6PC, G6PT is a ubiquitously expressed G6P/Pi antiporter (Lin et al., 1998; Chen et al., 2008). The known human G6PT variants and their functional impacts have been described (Hiraiwa et al., 1999; Chen et al., 2000; Chen et al., 2002).

**FIGURE 3:**
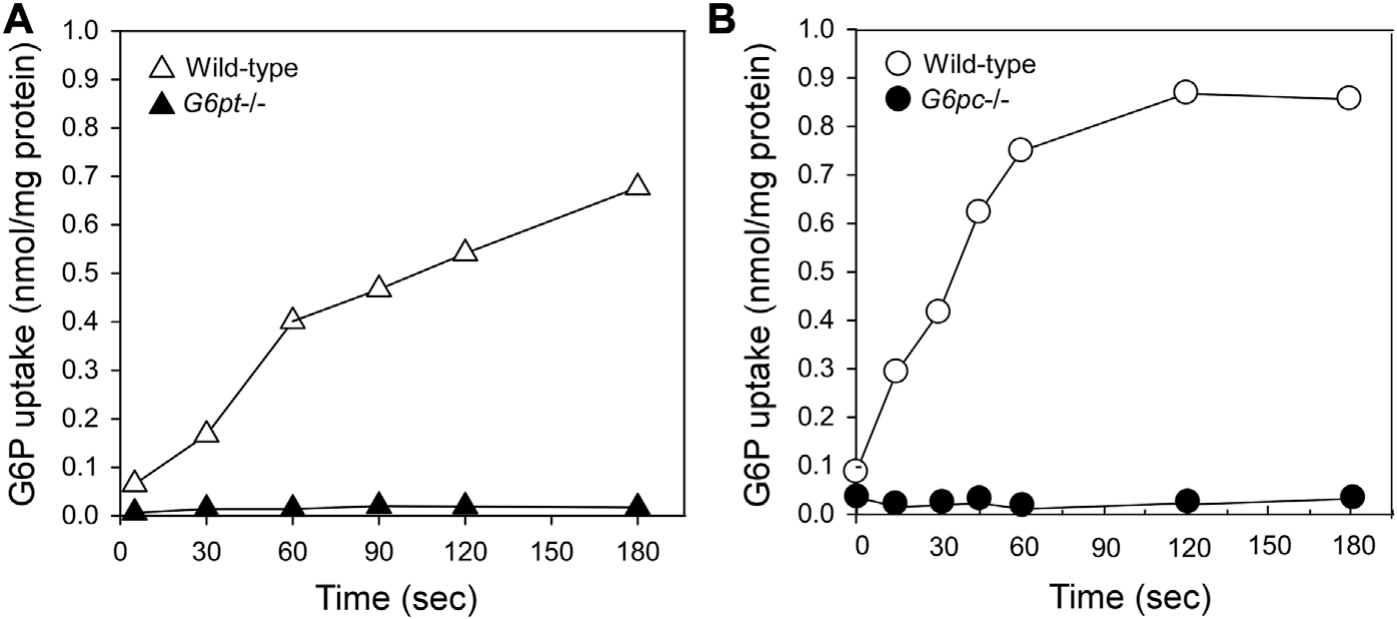
G6Pase-α and G6PT are functionally co-dependent. Microsomal G6P uptake was measured using [U^14^C] G6P (Lei et al., 1996; Chen et al., 2003). Intact hepatic microsomes from WT mice transport G6P efficiently (Lei et al., 1996; Chen et al., 2003). **(A)** Intact hepatic microsomes from the *G6pt*−/− mice, that lack G6PT but have a functional G6Pase-α, have no significant G6P uptake (Chen et al., 2003). **(B)** Intact hepatic microsomes from the *G6pc*−/− mice, that lack G6Pase-α but have a functional G6PT protein also have no significant G6P uptake (Lei et al., 1996). Therefore, G6Pase-α activity is required for G6P transport into the microsomes, and G6Pase-α and G6PT are functionally co-dependent.

The catalytic center of G6Pase-α lies in the lumen of the ER (Ghosh et al., 2002) (Figure 1). Therefore, endogenous glucose production by G6Pase-α depends upon transport of the cytoplasmic G6P into the ER lumen by G6PT, the rate-limiting step in G6P hydrolysis. There is a strong co-dependence between G6Pase-α and G6PT. Intact liver microsomes from wild-type mice transport G6P efficiently (Figure 3) (Lei et al., 1996; Chen et al., 2003). As expected, intact liver microsomes from *G6pt*−/− mice, that lack G6PT but have a functional G6Pase-α, have no significant G6P uptake (Figure 3A) (Chen et al., 2003). However, intact liver microsomes from G*6pc*−/− mice, that lack G6Pase-α but have a functional G6PT protein, also have no significant G6P uptake (Figure 3B) (Lei et al., 1996), indicating that G6Pase-α activity is required for G6P transport into the microsomes, and G6Pase-α and G6PT are functionally co-dependent (Lei et al., 1996). Since G6PT is the only antiporter that can couple to G6Pase-α α (Chen et al., 2008; Pan et al., 2011), it is likely a physical interaction between the proteins is important to the functional coupling.

**FIGURE 4:**
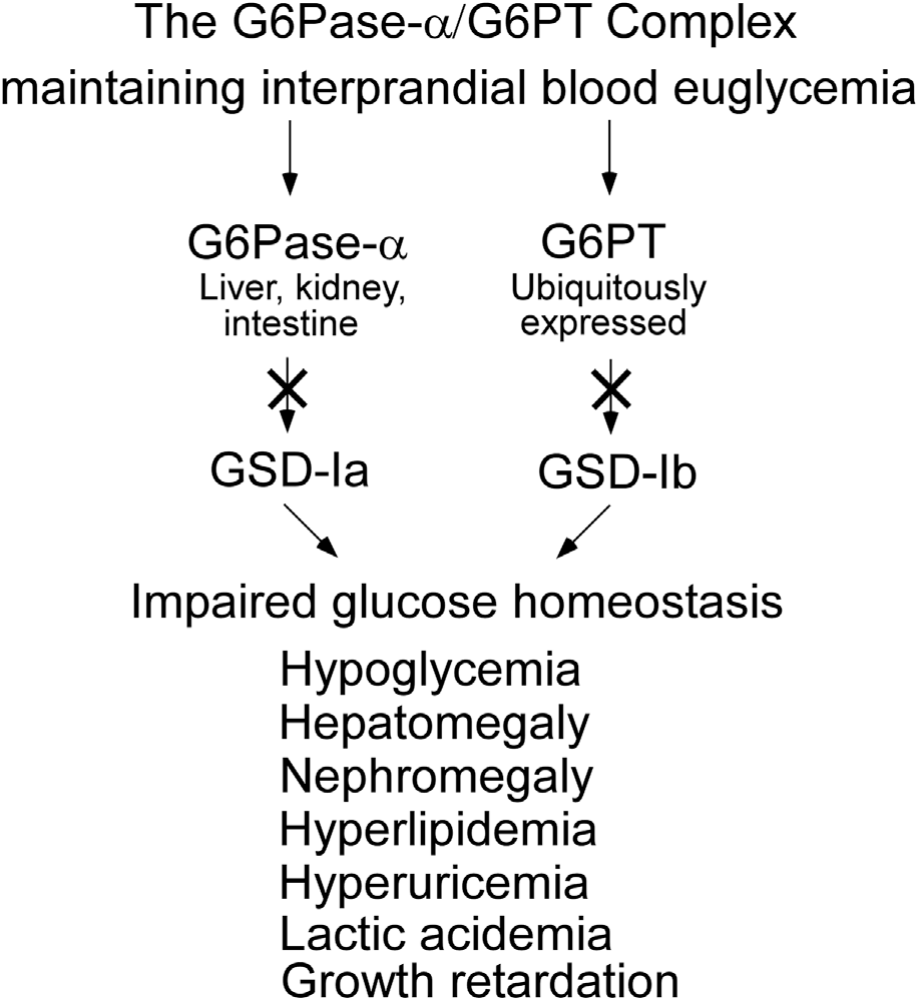
GSD-Ia and GSD-Ib share a common metabolic phenotype. The G6Pase-α enzyme, deficient in GSD-Ia, and the G6PT protein, deficient in GSD-Ib, form a complex that is required to maintain interprandial blood euglycemia. A pathogenic variant in either disrupts the function of the complex leading to a common metabolic phenotype, whose initial presentation is impaired blood glucose homeostasis.

**FIGURE 5:**
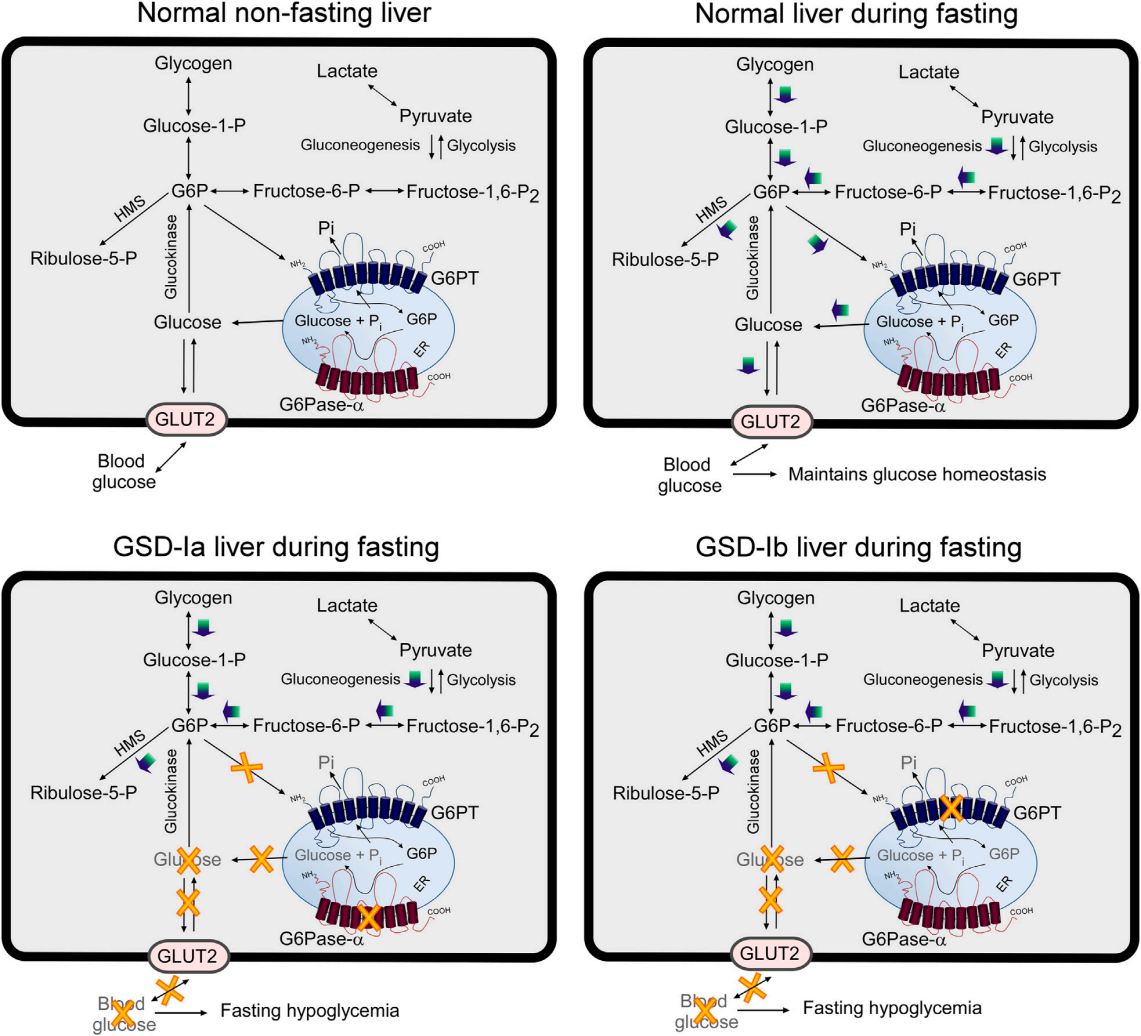
Pathways for G6P metabolism in normal, GSD-Ia, and GSD-Ib liver. During fasting, G6P, the end product of gluconeogenesis and glycogenolysis, is transported from the cytoplasm into the lumen of the ER by G6PT. Inside the ER, G6P is hydrolyzed by G6Pase-α and the resulting glucose is transported back into the cytoplasm then released into the circulation to maintain blood glucose homeostasis. In the GSD-Ia liver, which lacks a functional G6Pase-α, the co-dependent G6PT loses activity (Lei et al., 1996) and cytoplasmic G6P is not transported efficiently into the ER. Any G6P resident in the ER cannot be converted to glucose, resulting in a failure to release glucose to the blood, leading to hypoglycemia following a short fast. In the GSD-Ib liver, which lacks a functional G6PT, G6P cannot be transported into the ER for glucose production by G6Pase-α, leading to hypoglycemia following a short fast (Chen et al., 2003). The GLUT2, responsible for the transport of glucose in and out of the cell, is shown embedded in the plasma membrane. G6PT and G6Pase-α are shown embedded in the ER membrane. HMS, hexose monophosphate shunt; GLUT2, glucose transporter 2.

As a result of the G6Pase-α/G6PT functional co-dependence, a defect in either G6Pase-α or G6PT disrupts endogenous glucose production from G6P. The physiological consequence is impaired glucose homeostasis for all GSD-I patients (Figure 4). In gluconeogenic organs, G6P participates in multiple metabolic pathways including: G6Pase-α/G6PT-mediated glucose production; glycolysis; the hexose monophosphate shunt (HMS) also known as the pentose phosphate pathway; and glycogen synthesis (Figure 5). The inability of GSD-I patients to hydrolyze G6P to glucose leads to the reprogramming of G6P metabolism, resulting in: increased glycogen accumulation; increased lipid synthesis; enhanced glycolysis; and increased activity of the HMS (Cho et al., 2018a; Gjorgjieva et al., 2018). These outcomes are reflected in the clinical manifestations seen in GSD-Ia and GSD-Ib, which include fasting hypoglycemia; hepatomegaly caused by excessive glycogen/neutral fat accumulation; nephromegaly caused by excessive glycogen accumulation; hyperlipidemia; hyperuricemia; and lactic acidemia (Figure 4) (Chou et al., 2002; Chou et al., 2010a; Chou et al., 2010b; Chou et al., 2015). The metabolic perturbations also contribute to hepatocellular adenoma/carcinoma (HCA/HCC) development in GSD-I (Farah et al., 2016; Cho et al., 2017; Cho et al., 2018a; Cho et al., 2018b; Gjorgjieva et al., 2018; Liu et al., 2021; Gautam et al., 2023).

There is no pharmacologic therapy approved by the U.S.A. Food and Drug Administration (FDA) for GSD-I. GSD-I infants ≤ age 6 months cannot produce sufficient pancreatic amylase to digest uncooked cornstarch and are typically supported by a nocturnal nasogastric drip of glucose to avoid hypoglycemia (Greene et al., 1976). For patients aged 6–12 months or older, there are two current dietary protocols. One consists of uncooked cornstarch, a slow-release carbohydrate, which can support euglycemia between meals (Chen et al., 1984). The second, an FDA approved hydrothermally processed high amylopectin cornstarch, Glycosade^®^ (Vitaflo International Ltd, Liverpool, United Kingdom) (https://glycosadeusa.com) that can maintain normoglycemia for 7–8 h, which is significantly longer than a single serving of uncooked cornstarch (Correia et al., 2008; Ross et al., 2020). When strictly adhered to, the dietary therapies are effective in promoting blood glucose homeostasis. However, the doses of uncooked cornstarch or Glycosade must be titered for each patient in both amount and frequency to minimize stomach complications, Furthermore the dietary regimes are difficult to adhere to and socially disruptive to maintain. Many GSD-Ib patients, who are prone to inflammatory bowel disease, are reported to discontinue Glycosade because it can increase abdominal pain, diarrhea, and flatulence (Ross et al., 2020). Moreover, the underlying pathological processes remain uncorrected in these metabolically compensated patients. Consequently, the severe long-term complications of GSD-I, namely, HCA/HCC and renal disease, still occur in metabolically compensated patients (Chou et al., 2002; Rake et al., 2002; Chou et al., 2010a; Chou et al., 2010b; Kishnani et al., 2014). In addition, GSD-Ib patients also manifest neutropenia and myeloid dysfunction, which have been extensively reviewed elsewhere (Visser et al., 2000; Visser et al., 2002).

In this review, we provide an overview of the preclinical genetic studies that primarily focused on recombinant adeno-associated virus (rAAV) vector-mediated gene augmentation therapies for GSD-Ia and GSD-Ib. We outline the phase I/II gene augmentation clinical trial for human GSD-Ia (NCT03517085) that was launched by Ultragenyx Pharmaceutical Inc. (Novato, CA) in 2018, resulting in a phase III (NCT05139316) initiated in 2021. We also briefly address other applicable genetic approaches, including transient mRNA augmentation that entered a phase I trial for GSD-Ia (NCT05095727) initiated by Moderna (Cambridge, MA) in 2022. We conclude with a brief review of preclinical studies using the CRISPR/Cas9-based *in vivo* genome editing technology to correct a prevalent pathogenic human G6PC variant in GSD-Ia.

## 2 Gene delivery systems and animal models

### 2.1 Recombinant adeno-associated virus vectors

The rAAV vectors have emerged as one of the most promising gene delivery vehicles for the treatment of a variety of human diseases (reviewed in Wang et al., 2019; Li and Samulski, 2020; Mendell et al., 2021). To date, over 150 clinical trials involving rAAV vectors are registered at ClinicalTrials.gov. In most cases, the therapeutic strategy involves gene augmentation, targeting a monogenic autosomal recessive disorder by delivering additional copies of the wild-type (WT) protein coding sequence.

The AAV has a single-stranded DNA genome of approximately 4.7 kb. At each end of the linear DNA are 145 b inverted terminal repeat (ITR) that flank two open reading frames (*rep* and *cap*). Both *rep* and *cap* can be replaced by a cargo gene, since only the ITRs are essential for genome replication and packaging (Carter, 1992; Hastie and Samulski, 2015). For efficient packaging, the recombinant genetic cargo is generally kept below 5.0 kb (Dong et al., 1996). All AAV serotypes can infect multiple tissues, with tissue tropism determined by the capsid serotype and host cell surface receptors (Michelfelder and Trepel, 2009; Huang et al., 2014). The rAAV vectors are often pseudotyped by cross packaging the ITR sequence of one AAV serotype, frequently AAV2, with the capsid of a different serotype (Wu et al., 2006; Kwon and Schaffer, 2008), such as AAV2/8 (AAV2 ITR, AAV8 capsid). This can improve targeted tissue transduction *in vivo*, minimize the risk of off target toxicity, and circumvent problems of preexisting immunity. The rAAV vectors rarely integrate into the host genome, remaining predominantly as circular episomal elements, that can establish long-term transgene expression (Li and Samulski, 2020; Mendell et al., 2021). Durable gene therapy depends upon minimizing loss of both the episomal transgene and its expression.

A major rate-limiting step in the infection cycle of AAV is the conversion of the single-stranded (ss) DNA genome into double-stranded (ds) DNA required for gene expression (Ferrari et al., 1996; Fisher et al., 1996). The self-complementary AAV (scAAV) vectors were developed to overcome this limitation by packaging the vector as a single inverted repeat sequence that can fold itself directly into dsDNA (McCarty et al., 2001; Wang et al., 2003). While the scAAV vectors can enhance transduction efficiency, a disadvantage is the consequent reduction in gene packaging capacity to <2.5 kb.

The essential components of the rAAV expression cassette consist of a promoter/enhancer, a therapeutic transgene, and a poly(A) tail. Strategies to increase the efficacy of the rAAV vector upon transduction, such as optimization of the promoter/enhancer, inclusion of an intron, codon optimization, codon substitution of the open reading frame of the transgene, use of synthetic poly(A) sequences, and strategies to minimize immune responses have been reviewed by Li and Samulski (2020). While AAV is consider a relatively weak activator of both innate and adaptive immunity when compared to vectors such as adenovirus (Mays and Wilson, 2011), the AAV capsid proteins and encoded transgene product can be the targets for host immune responses. Strategies to minimize the immunological responses in clinical rAAV gene therapy have been extensively reviewed (Verdera et al., 2020; Muhuri et al., 2021; Weber, 2021). Since AAV transduction is not tissue-specific there are ongoing investigations to increase tissue-specific targeting (Powell et al., 2015; Rodríguez-Márquez et al., 2021). A further area of focus is to prolong hepatic expression of the episomal AAV transgene, which is subject to mitotic dilution (Piccolo et al., 2021; Nathwani et al., 2022).

### 2.2 Animal models for GSD-Ia and GSD-Ib

There is one naturally occurring dog, and several transgenic mouse models for GSD-Ia. The GSD-Ia dog (Brix et al., 1995; Kishnani et al., 2001), generated by crossbreeding a carrier Maltese, heterozygous for the G6PC-p.M121I pathogenic variant, with a beagle, manifests all the typical symptoms of human GSD-Ia. The dogs display a severe metabolic phenotype and die at birth in the absence of dietary support. While the GSD-Ia dog is a valuable translational model for clinical development, its animal husbandry is particularly demanding, and compared to mouse models is slower to reach maturity, has a smaller litter size, and a longer lifespan. The GSD-Ia dog is therefore best suited to late-stage preclinical studies validating preclinical data obtained in mice.

The global *G6pc*-deficient (*G6pc*−/−) mouse line was generated by replacing exon 3 and the associated introns of the murine *G6pc* gene with a neomycin cassette (Lei et al., 1996). The *G6pc*-R83C mouse line, expressing a human *G6PC* pathogenic null variant p.R83C, was generated by CRISPR/Cas9-mediated gene editing (Arnaoutova et al., 2021). Both *G6pc*−/− and *G6pc*-R83C mice manifest all symptoms of human GSD-Ia, namely, hypoglycemia, growth retardation, hepatomegaly, nephromegaly, hyperlipidemia, hyperuricemia, and mild lactic acidemia. If not maintained on a strict glucose therapy, the mice rarely survive weaning and live no more than 3 weeks, mimicking the lethality seen with untreated human GSD-Ia patients. Notably, human GSD-Ia patients (Chou et al., 2002; Chou et al., 2010a) and the GSD-Ia dog (Brix et al., 1995; Kishnani et al., 2001) exhibit a more marked lactic acidosis than the GSD-Ia mice.

Studies of long-term disease complications with the *G6pc*−/− (Lei et al., 1996) or the *G6pc*-R83C (Arnaoutova et al., 2021) mouse line are very difficult, requiring labor-intensive glucose therapy. In addition, neither of the mouse lines can be subjected to fasting glucose studies. To overcome these restrictions, two independent transgenic mouse lines (*G6pc* ^*fx/fx*^) harboring a latent conditional null allele for *G6pc* were created by flanking exon 3 of the *G6pc* gene with *loxP* sites (Peng et al., 2009; Mutel et al., 2011). Liver-specific *G6pc*-deficient (L-*G6pc*−/−) mice were then generated by the CRE-lox strategy, crossing the *G6pc* ^*fx/fx*^ mice with mice expressing a tamoxifen-dependent Cre-recombinase under the control of the liver-specific serum albumin promoter (Schuler et al., 2004). The L-*G6pc*−/− mice are viable, exhibit normoglycemia in the fed state, and manifest a liver phenotype mimicking that of human GSD-Ia. Importantly, they can sustain 24 h of fasting, chiefly *via* endogenous glucose production provided by the kidney and intestine which still possess the WT G6Pase-α (Mutel et al., 2011; Cho et al., 2017). The L-*G6pc*−/− mice develop HCA/HCC at age 72–78 weeks with an incidence of ∼100% (Mutel et al., 2011; Cho et al., 2017; Cho et al., 2019).

Using the L*-G6pc*−/− mice, we and others have investigated the molecular mechanisms underlying HCA/HCC development in GSD-I. The reprogramming of hepatic G6P metabolism (Cho et al., 2018a; Gjorgjieva et al., 2018) mentioned above also leads to ER stress and altered expression of tumor suppressors (Gjorgjieva et al., 2018), while the marked glycogen accumulation can initiate malignant transformation of the liver *via* activation of the YAP (Yes-associated protein) signaling pathway (Liu et al., 2021). The hepatosteatosis in GSD-I downregulates Sirtuin 1 signaling, leading to impaired hepatic autophagy (Farah et a., 2016; Cho et al., 2017; Gautam et al., 2023) and mitochondrial dysfunction (Cho et al., 2018b; Gjorgjieva et al., 2018). Together these contribute to HCA/HCC development in GSD-I.

While the tamoxifen-mediated *G6pc* gene excision is efficient, the L-*G6pc*−/− mice still express residual hepatic G6Pase-α activity (Cho et al., 2017; Cho et al., 2019). The WT mice express 180.4 ± 9.1 units. The residual hepatic G6Pase-α activity in 76-week-old L-*G6pc*−/− mice (n = 14) ranged from 1.7 to 7.5 units with an average value of 4.1 ± 0.4 units, representing 2.3% of normal hepatic G6Pase-α activity. Among the 14 L-*G6pc*−/− mice, 13 (93%) developed HCA. The mechanism underlying the high incidence of HCA/HCC in the L-*G6pc*−/− mice is unclear, but it clearly depends on the specific absence of liver G6PC. So, while the L-*G6pc*−/− mice are an excellent model to study long-term complications of hepatic G6Pase-α deficiency, they are inappropriate for developing therapies to address the initial abnormal metabolic phenotype.

There are no known large animal models for GSD-Ib. A global *G6pt* deficient (*G6pt*−/−) mouse line was generated by replacing exon 1 and the flanking intron 1 of the murine *G6pt* gene with a neomycin cassette (Chen et al., 2003). The *G6pt*−/− mice manifest all the metabolic and myeloid defects of human GSD Ib, namely, hypoglycemia, growth retardation, hepatomegaly, nephromegaly, hyperlipidemia, hyperuricemia, mild lactic acidemia, neutropenia, and myeloid dysfunction. If left untreated, the *G6pt*−/− mice rarely survive weaning and live no more than 3 weeks, modeling the lethality seen with untreated human GSD-Ib patients.

To study long-term complications in GSD-Ib, a transgenic mouse line (*G6pt* ^*fx/fx*^) harboring a latent conditional null allele for *G6pt* was generated by flanking exons two to five of the *G6pt* gene with *loxP* sites (Raggi et al., 2018). Liver-specific *G6pt*-deficient (L-*G6pt*−/−) mice were generated by the CRE-lox strategy, crossing the *G6pt* ^*fx/fx*^ mice with mice expressing a tamoxifen-dependent Cre-recombinase under the control of the liver-specific serum albumin promoter (Schuler et al., 2004). The L-*G6pt*−/− mice sustain fasting, reach adulthood, develop HCA/HCC, and manifest a liver phenotype mimicking that of human GSD-Ib (Raggi et al., 2018) while maintaining WT G6PT expression in the kidney and intestine.

## 3 Gene therapy for GSD-Ia

### 3.1 Gene therapy for GSD-Ia using the rAAV vectors

The G6Pase-α proteins are highly conserved across the human, mouse, rat, and dog, sharing 87%–91% amino acid sequence identity, and a conserved amino acid sequence in their catalytic centers (Chou et al., 2002; Chou et al., 2010a; Chou et al., 2015). Several promoters, enhancers, and gene constructs in combination with different AAV serotypes have been investigated in GSD-Ia therapy. The gene constructs included mouse, dog, and human cDNAs encoding G6Pase-α directed by the chicken β-actin (CBA) promoter/cytomegalovirus (CMV) enhancer (Ghosh et al., 2006); the canine *G6PC* gene promoter/enhancer (Koeberl et al., 2006); a minimal human *G6PC* promoter/enhancer (miGPE) (Koeberl et al., 2008); and an extended human *G6PC* promoter/enhancer (GPE) (Yiu et al., 2010). Both ssAAV and scAAV vectors have also been investigated. The studies have yielded valuable translational information. Firstly, the human, canine and murine *G6PC* encoded proteins are functionally equivalent. Each is enzymatically active when expressed in the *G6pc*−/− mouse liver and each couples functionally with murine G6PT to alleviate the metabolic abnormalities of GSD-Ia. Secondly, the GPE promoter (rAAV8-GPE-G6PC) is significantly more efficient in directing persistent transgene expression in the liver of *G6pc*−/− mice, compared to the CBA promoter (rAAV8-CBA-G6PC) (Yiu et al., 2010). In addition, the use of the CBA promoter/CMV enhancer elicits hepatic CD8^+^ lymphocyte infiltration which correlates with a rapid decline in *G6PC* transgene expression and the reduction in longer-term efficacy (Yiu et al., 2010). Most of these studies have been extensively reviewed by Chou and Mansfield (2011) and Jauze et al. (2019), and will not be covered here. This section focuses on the development of the clinical rAAV8-GPE-G6PC vectors.

#### 3.1.1 Development of the clinical rAAV-G6PC vector

In translating preclinical mouse studies into the clinic, minimizing vector dose while maximizing therapeutic efficacy will reduce patient exposure to the virus. Three complementary approaches were used to address this: optimization of gene expression through selection of the gene promoter/enhancer sequences; codon-optimization of the native *G6PC* coding sequence to improve translation; and amino acid substitution in the coding sequence to increase the specific activity of the encoded G6Pase-α enzyme. The AAV2/8 serotype was selected for its well-characterized efficiency in transducing the liver (Thomas et al., 2004; Davidoff et al., 2005).

The small size of the *G6PC* coding region (1074 bp) offers considerable design flexibility in optimizing the clinical rAAV vector. In early genetic characterization of the human *G6PC* gene structure and function, Lin et al*.* (1997) had mapped the minimal (mi) proximal *G6PC* promoter/enhancer elements (GPE) to the region stretching from the translation initiation codon ATG to −382 nucleotides upstream, which contains hepatocyte nuclear factor 1 and 3 binding motifs, cAMP response elements, insulin response elements, and a TATA box. Koeberl et al. (2008) constructed scAAV8**-**miGPE-G6PC, a self-complementary rAAV8 vector expressing human G6PC under the control of miGPE. When this vector was infused into 2-week-old *G6pc*−/− mice at 1 x 10^13^ vp/kg, the results showed that at age 26 weeks, the treated mice had restored hepatic G6Pase-α activity to WT levels as measured by quantitative phosphohydrolase assays (Koeberl et al., 2008). However, a histochemical analysis of G6Pase-α activity showed only a low level of expression throughout the hepatocytes compared to WT mouse livers, contradicting the quantitative assay. The reason for the discrepancy between the assays is unclear. One possible explanation is the quantitative assays used by Koeberl et al. (2008) had a low sensitivity, due to a background non-specific hydrolase activity ∼14% of the control activity. The gold standard for quantitative phosphohydrolase assays is the use of isolated liver microsomes to reduce the non-specific hydrolase background activity to less than 1% of the control activity.

While the scAAV vectors can express a gene more rapidly and more strongly than the ssAAV constructs, there is a trade-off in cargo size. To determine if there were additional critical gene control elements further upstream of the proximal promoter that could further enhance gene expression, sequences from nucleotides −382 to −2,864 of the human *G6PC* gene region, which contained a number of additional transcription factor binding sites were examined. Yiu et al. (2010) constructed ssAAV8-GPE-G6PC, a single-stranded vector expressing human *G6PC* controlled by the −2,864 to −1 nucleotide region of the human *G6PC* promoter. When this vector was infused into 2-week-old *G6pc*−/− mice at 1.5 x 10^13^ vp/kg, it restored hepatic G6Pase-α activity to greater than or equal WT levels at age 24 weeks, as estimated both by a gold standard quantitative phosphohydrolase assay using the isolated hepatic microsomes and the histochemical assay. These animals also exhibited normal levels of blood glucose, blood metabolites, hepatic glycogen, and hepatic fat, and could sustain 6 h of fasting (Yiu et al., 2010).

Both ssAAV8-GPE-G6PC and scAAV8-miGPE-G6PC studies infused 2-week-old *G6pc*−/− mice from the same source, used an AAV2/8 vector at equivalent doses (1-1.5 x 10^13^ vp/kg), and showed good efficacy in treating the *G6pc−/−* mice, but the vectors had been manufactured and tested in different laboratories. Since the ssAAV8-GPE-G6PC vector (Yiu et al., 2010) was developed at the National Institutes of Health (NIH) and the scAAV8-miGPE-G6PC vector (Koeberl et al., 2008) was developed at Duke University, a collaborative comparative study was undertaken to select the most effective construct. For this study, the two vectors were amplified and purified at an independent vector facility, the University of Florida Powell Gene Therapy Center Vector Core Laboratory (Gainesville, FL). A common set of experimental protocols were established by mutual agreement between Duke and NIH and the side-by side studies of both vectors were conducted independently at each center. All viral transductions were performed on 2-week-old *G6pc*−/− mice, and the efficacy evaluated at age 12 weeks (Lee et al., 2013). The results showed that the ssAAV8-GPE-G6PC vector directed 3.5-fold higher levels of hepatic G6Pase-α expression, achieved greater reduction in hepatic glycogen accumulation, and led to a better tolerance of fasting, compared to the scAAV8-miGPE-G6PC vector. As a result, the ssAAV8-GPE-G6PC vector was selected for clinical translation in human GSD-Ia. Since the studies described below are all directed by rAAV2/8 with the native human *G6PC* promoter/enhancer (GPE) expressing WT human G6PC protein sequence, we will abbreviate the construct name from ssAAV8-GPE-G6PC to rAAV-G6PC-WT for the rest of this review article.

#### 3.1.2 Strategies to increase the expression and catalytic activity of the G6PC transgene

Codon optimization, that retains the native amino acid sequence of a protein, is widely used to increase the translation efficiency of gene constructs (Foster et al., 2008; Ward et al., 2011). Therefore, a codon-optimized (co) human G6PC with 20% change in the native *G6PC* coding sequence, designated rAAV-coG6PC, was constructed and tested (Kim et al., 2017b). The rAAV-coG6PC vector yielded 2-fold higher expression than the rAAV-G6PC-WT vector, resulting in a 50% reduction in viral dose to restore WT hepatic G6Pase-α activity. Extensive preclinical characterization of GSD-Ia gene therapies in the mouse and canine models, using both the rAAV-G6PC-WT and rAAV-coG6PC vectors, established gene therapy for GSD-Ia as being safe and efficacious (Yiu et al., 2010; Lee et al., 2012; Kim et al., 2015; Lee et al., 2015; Kim et al., 2017a; Kim et al., 2017b; Lee et al., 2018).

Another approach to optimize dose efficacy for an enzyme, like G6Pase-α, is to increase the specific activity of the enzyme. *In vitro* expression assays have routinely shown that the canine G6Pase-α isozyme is significantly more active than human G6Pase-α (Zhang et al., 2019). Sequence analysis of all *G6PC* genes across the evolutionary tree identified several amino acid candidates that might improve the specific activity of the human enzyme. Upon testing candidate variants, a Ser-298 to Cys-298 substitution naturally found in dog, mouse, rat, and several primate G6Pase-α isozymes, stood out. When incorporated into the WT human G6Pase-α sequence, S298C markedly enhanced enzymatic activity (Zhang et al., 2019). Structure-function studies have previously shown that the structural integrity of the transmembrane helices is important for G6Pase-α enzyme activity (Shieh et al., 2002). Consistent with this, the G6Pase-α-S298C variant, that lies in transmembrane helix-8 (Figure 1), was shown to increase enzyme stability (Zhang et al., 2019).

**FIGURE 6:**
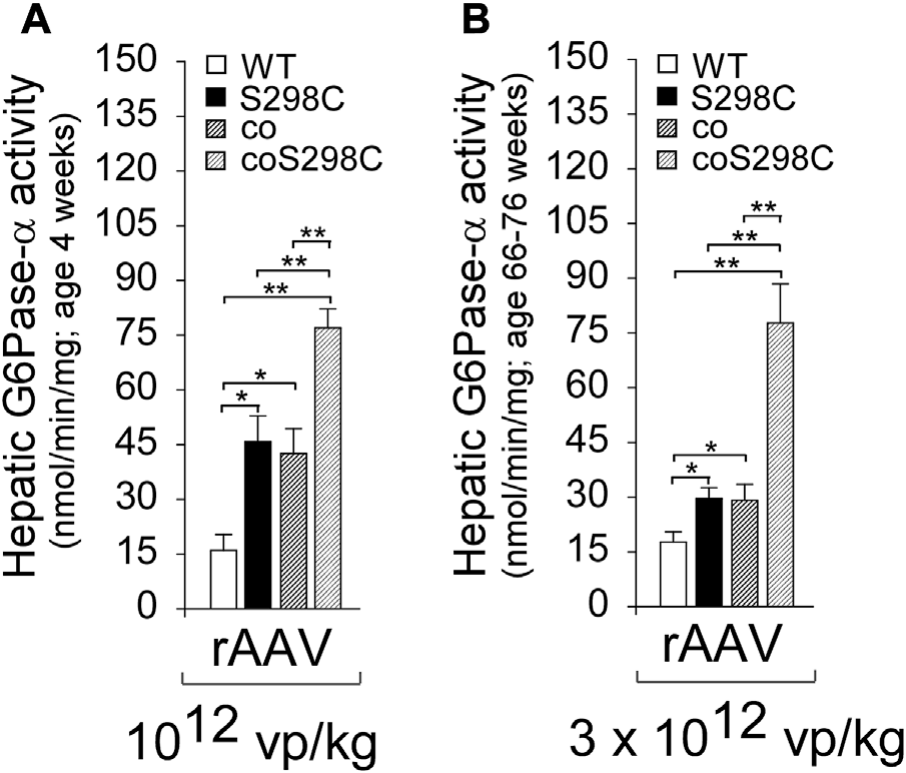
The effects of codon optimization and catalytic activity optimization are additive and stable. **(A)** *G6pc* ^_/_^ mice were treated at age 2 weeks with 10^12^ vp/kg rAAV-G6PC-WT, rAAV-G6PC-S298C, rAAV-coG6PC, or rAAV-coG6PC-S298C (n = 6 per group) and analyzed at age 4 weeks (Zhang et al., 2019). **(B)** *G6pc* ^_/_^ mice were treated at age 2 weeks with 3 x10^12^ vp/kg rAAV-G6PC-WT, rAAV-G6PC-S298C, rAAV-coG6PC, or rAAV-coG6PC-S298C (n = 6 per group) and analyzed at age 66–76 weeks. Hepatic microsomal G6Pase-α activity shows the additive effects of the codon and catalytic activity optimizations.

To understand if codon optimization and amino acid substitutions had additive effects on expression, Zhang et al. (2019) performed transient expression assays, comparing the enzymatic activity of the constructs: G6Pase-α-WT, G6Pase-α-S298C, coG6Pase-α, and coG6Pase-α-S298C. Both G6Pase-α-S298C and coG6Pase-α gave a similar 2-fold increase in activity compared to G6Pase-α-WT, and when combined in coG6Pase-α-S298C provided a further additive increase to 4-fold G6Pase-α-WT activity (Zhang et al., 2019). This outcome was confirmed in a short-term (4 weeks) *in vivo* gene transfer study in *G6pc*−/− mice using these rAAV8 vectors at 10^12^ vp/kg. Hepatic G6Pase-α activities in *G6pc−/−* mice treated with rAAV-G6PC vectors containing S298C, coG6PC, or coG6PC-S298C were 2.9-, 2.7-, and 4.8-fold higher, respectively, than that in rAAV-G6PC-WT-treated mice (Figure 6A).

A long-term (66–76 weeks) efficacy study of these vectors was undertaken in *G6pc*−/− mice dosed at 3 x 10^12^ vp/kg. All treated *G6pc*−/− mice survived to age 66–76 weeks, and again the outcomes were additive, with hepatic G6Pase-α activities for S298C, coG6PC, or coG6PC-S298C being1.7-, 1.7-, and 4.4-fold higher, respectively, than in rAAV-G6PC-WT-treated mice (Figure 6B).

These studies provided two important outcomes and offered alternative strategies for clinical translation. Firstly, combining the S298C substitution with codon optimization, displayed 4-fold higher expression *in vivo* compared to the G6PC-WT construct. So, a 4-fold lower dose of vector could be used to obtain equivalent clinical results, providing the benefits of reduction in potential toxicities and immune activation. Secondly, the *G6PC*-S298C variant requiring only a 2-bp (0.2%) change to the *G6PC-*WT coding sequence could confer equal efficacy to the codon optimization approach, that changes 20% of the *G6PC-*WT coding sequence. While routinely used in clinical therapies, codon-optimized vectors may not always be optimal. Several studies have noted that broad changes in the native gene coding sequence, while maintaining the protein sequence, can potentially change RNA and DNA protein binding sites, impact RNA secondary structure, affect protein conformation and function, and alter post-transcriptional modifications that may reduce potency or efficacy (Stergachis et al., 2013; Mauro and Chappell, 2014; Bazzini et al., 2016; Rapino et al., 2018). To date, the long-term efficacy of codon optimization compared to non-optimized constructs in human clinical outcomes is not well characterized. While the commercial decision for GSD-Ia was to develop the traditional approach of a codon-optimized vector, both the rAAV-G6PC-S298C and rAAV-coG6PC-S298C vectors offer attractive clinical alternatives.

#### 3.1.3 Long term efficacy, HCA/HCC risk, and minimal therapeutic level of G6Pase-α activity

Longer-term (60–90 weeks) gene therapy studies in *G6pc*−/− mice have been conducted to evaluate the therapeutic value of the rAAV-G6PC vectors (Lee et al., 2012; Kim et al., 2015; Lee et al., 2015; Kim et al., 2017a; Kim et al., 2017b; Zhang et al., 2020). The studies clearly show that there are no observable long-term differences in pathophysiology between the WT, S298C, coG6PC, or coG6PC-S298C transduced *G6pc*−/− mice, when titered to express equivalent microsomal G6Pase-α enzymatic activity. Therefore, for the rest of this review, *G6pc−/−* mice, treated with any of these vectors, are collectively named the AAV-*G6pc*−/− mice**.**

Restoration of hepatic G6Pase-α activity in *G6pc*−/− mice can be reproducibly titrated by vector dose, from 1% to over 100% of control (*G6pc*+/+ and *G6pc*+/−) mouse hepatic G6Pase-α activity. To identify the minimum effective therapeutic dose, the phenotype of 72 AAV-*G6pc*−/− mice that lived over age 60 weeks and expressed 1%–63% of normal hepatic G6Pase-α activity were evaluated (Lee et al., 2012; Kim et al., 2015; Lee et al., 2015; Kim et al., 2017a; Kim et al., 2017b; Zhang et al., 2020). All 72 AAV-*G6pc*−/− mice maintained glucose homeostasis, sustained 24 h of fasting, displayed no detectable anti-G6Pase-α antibodies, and were protected against age-related obesity and insulin resistance. Forty-seven AAV-*G6pc*−/− mice expressing 3%–63% of normal hepatic G6Pase-α activity showed no evidence of HCA/HCC. However, among the 25 AAV-*G6pc*−/− mice expressing <3% of normal hepatic G6Pase-α activity, four (16%) developed HCA/HCC, establishing that 3% normal hepatic G6Pase-α activity is the threshold for tumor prevention. The studies also showed that full restoration of normal hepatic G6Pase-α activity is not required to confer significant therapeutic benefits in GSD-Ia therapy.

**FIGURE 7:**
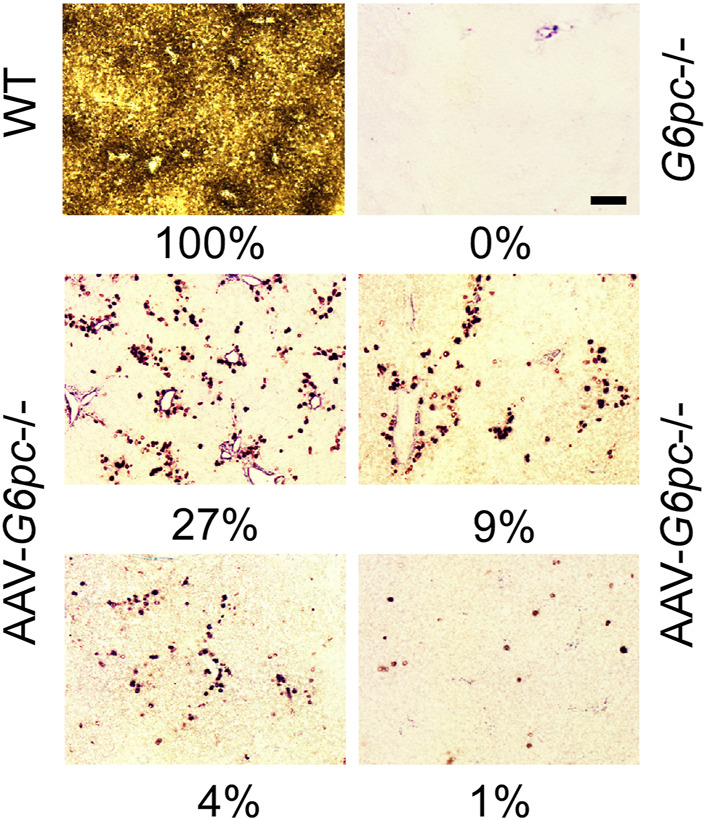
Histochemical analysis of hepatic G6Pase-α activity in WT, *G6pc*−/−, and *G6pc*−/− mice treated with rAAV-G6PC-S298C (AAV-*G6pc*−/−) (Zhang et al., 2020). Liver samples from control and rAAV-G6PC-S298C-treated mice were collected at sacrifice following 12 or 24 h of fast. In WT mice, G6Pase-α was distributed throughout the liver with significantly higher levels in proximity to blood vessels. The numbers in percentage represent hepatic microsomal G6Pase-α activity restored in the AAV-*G6pc*−/− mice. Scale bar = 20 µm.

Why is reconstitution of such a low level (≥3%) of normal hepatic G6Pase-α activity sufficient for AAV-*G6pc*−/− mice to maintain blood glucose homeostasis and prevent HCA/HCC development? One reason is that G6Pase-α is an enzyme catalyst, not a structural protein. Another consideration is that glucose homeostasis is regulated in the liver by functional zonation. Hepatocytes perform a wide range of functions that characterize the three discrete hepatocyte zones - periportal Zone 1, midzone Zone 2, and perivenous Zone 3 (Paris and Henderson, 2022) - that have different nutrient and oxygen status. G6Pase-α is primarily expressed in periportal Zone 1 (Jonges et al., 1990), which performs gluconeogenesis, beta-oxidation, protein/urea synthesis, and lipid metabolism (Paris and Henderson, 2022). Consistent with this, our enzyme histochemical analysis showed that in WT mice, G6Pase-α was distributed throughout the liver with foci of increased G6Pase-α activity in the periportal Zone 1 hepatocytes (Figure 7). As expected, G6Pase-α activity was not detectable in the liver sections of the *G6pc*−/− mice (Figure 7). This pattern of distribution, containing foci around blood vessels was also observed in the AAV-*G6pc*−/− mice (Figure 7), although the total activity staining was substantially weaker than WT. The periportal Zone 1 hepatocytes may not represent the majority of total liver G6Pase-α activity, but they are the best positioned to respond to rapid demands of blood glucose uptake or hepatic glucose release to maintain blood glucose homeostasis. Therefore, it is reasonable to suggest that restoring sufficient levels of ≥3% of normal G6Pase-α in Zone 1 hepatocytes is most important for GSD-Ia. Understanding the roles of the non-Zone 1 G6Pase-α in healthy and GSD-I liver requires further investigation.

Of interest, when the 60–90 week-old AAV-*G6pc*−/− mice that expressed 1%–63% of normal hepatic G6Pase-α activity were compared to age-matched WT mice, the AAV-*G6pc*−/− mice were protected against age-related obesity and insulin resistance that occur in WT mice (Kim et al., 2015; Kim et al., 2017b). This suggests that restoring hepatic G6Pase-α activity to levels below 100% of normal activity may have advantages. The mechanism of this remains to be understood but may be related to the calorie restriction that AAV-*G6pc*−/− mice have been shown to live under (Kim et al., 2015). The rAAV8 vector-mediated G6Pase-α transgene expression is primarily targeted to the liver and very little transgene expression is observed in the kidney and intestine (Lee et al., 2013). While liver is the main glucose producing organ during post-absorptive conditions, endogenous glucose can be produced by all three gluconeogenic organs, liver, kidney, and intestine to maintain blood euglycemia during a fast. In the absence of endogenous glucose production from the kidney and intestine, the AAV-*G6pc*−/− mice expressing 1%–63% of normal hepatic G6Pase-α activity elevate sirtuin 1 signaling, the positive mediator of calorie restriction (Ruderman et al., 2010).

#### 3.1.4 The AAV-G6pc−/− mice express increased hepatic G6P transporter (G6PT) activity

The rate-limiting step in endogenous glucose production by the G6Pase-α/G6PT complex is the G6PT-mediated microsomal uptake of G6P (Lei et al., 1996; Hiraiwa et al., 1999). Kim et al. (2015, 2017b) showed that in the presence of a reduced hepatic G6Pase-α activity in the AAV-*G6pc*−/− mice, there appears to be a partial compensatory mechanism that can increase G6PT expression. This enables the AAV-*G6pc*−/− mice expressing ≥1% of normal hepatic G6Pase-α activity to produce sufficient endogenous glucose to maintain euglycemia during prolonged fasts.

#### 3.1.5 Gene therapy in canine GSD-Ia using the rAAV vectors

Early studies treating newborn GSD-Ia dogs with either AAV-miGPE-G6PC (Koeberl et al., 2008) or AAV-CBA-G6PC (Weinstein et al., 2010) vector had prolonged survival for over 11 months. Two long-term follow-up studies using either rAAV-miGPE-G6PC with different serotypes (Brooks et al., 2018) or rAAV8-GPE-G6PC (Lee et al., 2018) showed that the occurrence of long-term complications differ markedly.

Brooks et al. (2018) showed that at age 4.1–8 years**,** four of the five rAAV-miGPE-G6PC-treated GSD-Ia dogs had HCA/HCC and all five exhibited progressive kidney disease with three developing renal failure. In contrast, Lee et al. (2018) showed that at age 5.8–7.1 years, none of the four rAAV-GPE-G6PC-treated GSD-Ia dogs had focal hepatic lesions or renal abnormalities. However, there were two significant design differences between these studies, the difference in the efficacy of the native promoter/enhancer being used and the nutritional support provided during therapy.

Comparing the two vectors used, Lee et al*.* (2013) showed that the rAAV-GPE-G6PC vector directs 3.5-fold more hepatic G6Pase-α expression than the rAAV-miGPE-G6PC vector. As a result, the miGPE study (Brooks et al., 2018) used from 1.8 to 7.0-fold lower initial doses and 23–70-fold less equivalent activity on follow up infusions, than the GPE study (Lee et al., 2018), which may explain, in part, the different outcomes reported.

In the rAAV-GPE-G6PC study (Lee et al., 2018), using the vector AAV2/8, two different administration protocols were used. In one protocol, two GSD-Ia dogs were treated at birth with 2 × 10^13^ vp/kg and found to require a boost of 2 × 10^13^ vp/kg, between ages 2 and 6 months to maintain euglycemia. In the other protocol, two GSD-Ia dogs were treated with a single infusion of 2 × 10^13^vp/kg at age 6 months. Unfortunately, intensive glucose nutritional support was maintained for the duration of the study, so while demonstrating vector safety, the study could not demonstrate efficacy. The authors did not clarify why nutritional support was maintained, or if there were any fasting challenges during the study.

In the rAAV-miGPE-G6PC study (Brooks et al., 2018), all 5 GSD-Ia dogs were treated at birth with 1–4 × 10^13^ vp/kg of an AAV2/8 or AAV2/9 construct followed by one to three additional infusions at 1-3 × 10^12^ vp/kg to support dogs when they could not maintain euglycemia. The additional infusions used up to three different vector pseudotypes (AAV2/9, AAV2/8, AAV2/7) in different combinations to avoid anti-AAV antibodies, and re-administrations ranged over varying periods of 2–58 months, for different dogs. The rationale for the selection of the vector pseudotype for each dog, for each infusion, or presence of anti-AAV antibodies was not provided, which could have given insights into vector safety and repeat infusion safety. In contrast to Lee et al. (2018) the treated GSD Ia dogs were maintained with a typical dog diet of three feedings per day, appropriate for testing efficacy. Survival of the dogs increased from a mean of 0.09 years in untreated dogs to a mean of 6.9 years in treated dogs. However, the lower doses and need to re-dose were not sufficiently informative to guide information on an effective clinical dose. Studies have shown that good metabolic control can prevent GSD-Ia patients from developing renal disease (Dambska et al., 2017; Okechuku et al., 2017), but the doses in the study of Brooks et al. (2018) were not effective in preventing death from renal failure and/or HCA/HCC.

#### 3.1.6 Clinical translation

**FIGURE 8:**
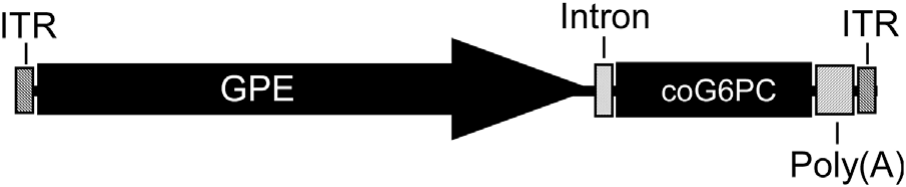
The clinical rAAV8-GPE-coG6PC construct. The AAV2/8 serotype contains the construct packaged in the AAV8 capsid. The construct consists of the AAV2 ITR flanking each end, with the human *G6PC* promoter/enhancer (GPE, −2,864 to −1 nucleotide region of the human *G6PC* gene), a synthetic intron (137 bp), the codon-optimized (co) human G6PC coding sequence (1074 bp), and a SV40 Poly(A) tail (∼240 bp). The overall size is 4.8 kb. Specific details of the intron and other sequences are provided in patents US 10113183 B2 and EP 3074510 B1.

Based on all the above, the rAAV-GPE-G6PC and rAAV-GPE-coG6PC vectors were licensed to Ultragenyx Pharmaceutical Inc., and in 2018 a phase I/II clinical trial (NCT03517085) of rAAV-GPE-coG6PC (DTX401) was initiated, that led to a longer-term follow-up study (NCT03970278). In 2022 a phase III clinical trial was initiated (NCT05139316). The final clinical construct is shown in Figure 8.

##### 3.1.6.1 Results of the phase I/II clinical study of human GSD-Ia (NCT03517085)

The clinical trial NCT03517085 has posted study results on the Clinicaltrials.gov website (Ultragenyx Pharmaceutical, 2022a). The trial enrolled 12 participants (4 female, 8 male) with GSD-Ia, ≥18 years of age, across six international sites, and studied 2 different doses of either 2 x 10^12^ GC/kg (cohort 1) or 6 x 10^12^ GC/kg (cohorts 2-4). The drug was delivered by intravenous IV) infusion of DTX401 with steroids (prednisone/prednisolone) to manage alanine aminotransferase elevation. Following infusion, a reactive steroid support regime was initiated, the difference in cohorts two to four being the steroid dose and timing. The primary endpoint was the number of Adverse Events, and their consequence, for the study length (week 52 or until 30 days following an early withdrawal). The secondary endpoint was the change from baseline in time to first hypoglycemic event over time. The published Study Results reports no participants withdrew from the study early and there was no dose-limiting toxicity. Four participants were affected by Serious Adverse Events: 2 in cohort 1 (metabolic disorder, migraine); 1 each in cohorts 2 (cellulitis) and 3 (lactic acidosis); and none in cohort 4. In the secondary outcome there was an overall improvement in glucose control measured by the time to first hypoglycemic event with cohort 1 showing a baseline of 4.4 ± 0.9 h increasing by an additional 4.2 ± 2.2 h, effectively maintaining euglycemia on average for 8 h. A longer-term follow-up presentation (Ultragenyx Pharmaceutical, 2022b; Riba-Wolman et al., 2022) reported that consistent with this, cohort 1 had decreased daily total cornstarch intake by −86% to −100% 3 years post-infusion, suggesting sustained glucose control had been established. Conclusions from the full cohort data await a peer-reviewed publication by the researchers and the outcomes of additional clinical trials to increase the number of participants treated. However, the Phase I/II NCT03517085 Study Results suggest that over 52 weeks the therapy can be safe and efficacious. A long-term follow-up study of these participants is in progress (NCT03970278).

##### 3.1.6.2 The phase III clinical study of human GSD-Ia (NCT05139316)

The phase III clinical trial NCT05139316 (Ultragenyx Pharmaceutical, 2022c), started in 2022, aims to enroll 50 participants 8 years and older with GSD-Ia in a placebo-controlled crossover study across 17 international locations. The participants will be divided into two cohorts of equal size. One cohort will receive an IV infusion of DTX401 and oral prednisolone, while the other will receive an IV infusion of saline and placebo oral corticosteroids. After 48 weeks, participants who received placebo will receive an IV infusion of DTX401, while the first treatment cohort will receive a placebo IV infusion of saline. The primary outcome measures will be the percent change from baseline to week 48 in daily cornstarch intake and the change from baseline to week 48 in the percentage of time spent in normal glucose control. Secondary outcomes will include a number of metabolic markers and a Glycogen Storage Disease Functional Assessment Diary (GSD FAD) Signs and Symptoms Scale.

#### 3.1.7 mRNA therapy using a codon-optimized G6PC-S298C variant

An mRNA augmentation therapy has recently been developed for GSD-Ia. Roseman et al. (2018), constructed several *G6PC* mRNA variants based on a thermostability design algorithm that predicted G6PC protein variants that might have increased intracellular expression and half-life. The lipid nanoparticle (LNP)-encapsulated *G6PC* mRNA variants were delivered as single systemic administrations at 1 mg/kg to the livers of L*-G6pc−/−* mice. The treated mice expressed an active G6Pase-α enzyme, displayed euglycemia along with reductions in liver mass, hepatic G6P, glycogen, triglycerides, and metabolic abnormalities associated with GSD-Ia. Notably, the mRNA was cleared from the liver after 24 h, although the G6PC protein diminished more slowly, over 12 days.

Cao et al. (2021) evaluated the efficacy of periodic mRNA therapy administration to correct metabolic abnormalities and prevent HCA/HCC using the L*-G6pc−/−* mice. Their synthetic mRNA, which contained complete N1-methylpseudouridine substitution, and included 5′and 3′untranslated regions along with the coding sequence and a poly(A) tail, was packaged in a LNP formulation and delivered intravenously. Supporting the study of Zhang et al. (2019), Cao et al. (2021) showed that the G6PC-S298C variant had a 2-fold higher specific activity than WT G6PC, when expressed *in vitro*, and the activity of the encoded enzyme was further increased by codon-optimization. As previously reported (Zhang et al., 2019), the increase in enzymatic activity of the G6PC-S298C variant correlated with a corresponding increase in the stability of the expressed protein, and notably, the overall clearance rate of G6PC-WT and G6PC-S298C proteins were similar.

From a safety perspective, the L-G6pc−/− mice treated *via* systemic administration of five consecutive weekly doses of 0.5 mg/kg of LNP-encapsulated *coG6PC*-S298C mRNA displayed no apparent increase in serum levels of IFNɣ, IL-1β, IL-6, TNFα, antibodies against the drug, or immune hypersensitivity (Cao et al., 2021). To address the long-term risk of HCA/HCC, L*-G6pc−/−* mice were pre-treated with a high fat/high sucrose diet to accelerate tumor development. The mice were then treated ten times with doses (0.25–0.5 mg/kg) of LNP-encapsulated *coG6PC*-S298C mRNA every 1–2 weeks *via* intravenous administration. While only one of 21 control mice developed a macroscopic lesion, 16 out of 26 (62%) untreated L-*G6pc*−/− mice developed HCA/HCC. Conversely, only 8 of 34 (24%) L-*G6pc*−/− mice treated with the *coG6PC*-S298C mRNA had visible lesions, suggesting that mRNA therapy decreased tumor burden (Cao et al., 2021).

Moderna (Cambridge, MA) launched a phase I clinical trial (NCT05095727) in 2022 to evaluate the safety and tolerability of mRNA-3745 (LNP-encapsulated co*G6PC*-S298C mRNA) and characterize the pharmacokinetic and pharmacodynamic response following intravenous administration of a single dose of mRNA-3745 to GSD-Ia patients. Intravenous dosing primarily delivers the mRNA to the liver. While a one to two weekly dosing regimen may be less attractive as a long-term therapeutic option, it could offer an effective gap intervention for the neonatal/pediatric population for whom rAAV-mediated gene therapy is known to be less effective due to the rapid growth of the liver and subsequent loss of vector to mitotic cell dilution (Cunningham et al., 2008; Yiu et al., 2010).

### 3.2 Gene editing for GSD-Ia

**FIGURE 9:**
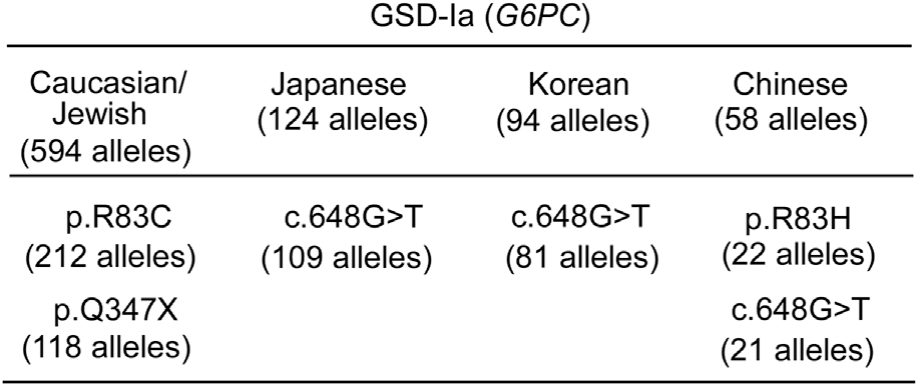
Ethnic differences in the prevalent G6PC variants identified in GSD-Ia patients (Fokkema et al., 2021) "https://databases.lovd.nl/shared/variants/G6PC?search_var_status=%3D%22Marked%22%7C%3D%22Public%22" accessed in March 2023. The total number of *G6PC* alleles genotyped for each ethnicity (Chou and Mansfield, 2008; Chou et al., 2010a; Chou et al., 2010b; Chou et al., 2017) is given in the heading. Only pathogenic variants with allele counts greater than 10 are listed.

The genetic construct in rAAV-mediated gene augmentation therapy is maintained episomally (Li and Samulski, 2020; Mendell et al., 2021). Currently, there is insufficient clinical data to understand if multi-decade episomal transgene expression can be maintained in human liver at a therapeutic level (Nathwani et al., 2022). The available data suggest transgene expression can be maintained in humans for at least 5 years (Nathwani and Tuddenham, 2020). However, one interpretation of the recent studies on the rate of physiological cell replacement in the mature human liver (Heinke et al., 2022) is that episomal gene augmentation might require re-administration to maintain efficacy over a lifetime. Genetic editing technologies that may be more durable than episomal expression are also being developed as GSD-Ia therapies. At present, these are designed to address individual pathogenic variants. Both CRISPR/Cas9 gene editing (Ran et al., 2013; Yang et al., 2016; Pankowicz et al., 2017; Schneller et al., 2017; Lino et al., 2018) and base editing technologies (Komor et al., 2016; Gaudelli et al., 2017; Nami et al., 2018; Ryu et al., 2018; Molla and Yang, 2019) are being developed to correct the more prevalent GSD-Ia variants (Chou and Mansfield, 2008; Chou et al., 2017) (Figure 9).

The CRISPR/Cas9 gene editing system (Ran et al., 2013; Yang et al., 2016; Pankowicz et al., 2017; Schneller et al., 2017; Lino et al., 2018) consists of two components, a Cas9 endonuclease and a single guide RNA (sgRNA) containing a gene variant-specific recognition domain. The Cas9/sgRNA can bind a genomic target that is proximal to a motif named the protospacer adjacent motif (PAM), and induce double-strand DNA breaks, which are then repaired by error-prone non-homologous end-joining or precise homology-directed repair. Using the *G6pc*-R83C mice, Arnaoutova et al. (2021) evaluated the efficiency of CRISPR/Cas9 gene editing technology to correct the pathogenic G6PC-p.R83C variant. They used a dual AAV8 delivery system. One vector contained the 503 bp *G6pc* donor sequence, the sgRNA directed by the U6 promoter and a TurboGFP marker protein driven by the CMV promoter. The second vector contained the *Staphylococcus aureus* (Sa) Cas9 directed by a liver-specific human thyroxine binding globulin (TBG) promoter and the sgRNA directed by the U6 promoter. The vectors were co-infused at a 10:1 ratio into newborn *G6pc*-R83C mice *via* the temporal vein. All treated newborn *G6pc*-R83C mice had their hepatic G6Pase-α activity restored and survived at least 16-week, while untreated mice could not survive (Arnaoutova et al., 2021). At ages 8 and 16 weeks, the treated *G6pc*-R83C mice expressed ≥3% of normal hepatic G6Pase-α activity, had a normal metabolic phenotype, and could survive a 24 h fast, consistent with the findings of the *G6pc* gene augmentation studies.

Base editing is a CRISPR/Cas9-based genetic technique that can precisely alter a single nucleotide within a target gene without inducing a double-strand DNA break that might trigger recombination events (Komor et al., 2016; Gaudelli et al., 2017; Nami et al., 2018; Ryu et al., 2018; Molla and Yang, 2019). The adenine base editors (ABE) (Gaudelli et al., 2017) use a fusion protein consisting of a catalytically impaired Cas9 (dCas9) fused to an adenosine deaminase that can edit an A•T base pair to a G•C base pair. A sgRNA directs the system to a specific base in the target gene, and the Cas9 nickase activity induces a single-strand DNA break at the targeted base. Importantly, base editors work in both dividing and non-dividing cells (Komor et al., 2016; Gaudelli et al., 2017; Nami et al., 2018; Ryu et al., 2018; Molla and Yang, 2019). Beam Therapeutics (Boston, MA) has generated two humanized GSD-Ia mouse models, hu*G6pc*-R83C and hu*G6pc*-Q347X carrying the G6PC-p.R83C and G6PC-p.Q347X variants, respectively. A collaborative study between the Chou group at the NIH and Beam Therapeutics, using the ABE editing system, showed that a single, systemic administration of BEAM-301, ABE mRNA/sgRNA packaged in a LNP formulation, to newborn hu*G6pc*-R83C mice mitigated fasting hypoglycemia and corrected metabolic abnormalities. If these therapeutic approaches are stable, address HCC/HCA formation and translate into successful clinical trials, they offer an additional variant-specific therapeutic option that may result in permanent restoration of endogenous G6Pase-α expression. Additional genome editing technologies, such as prime editing (Anzalone et al., 2020), that can create substitutions, insertions, and/or deletions over a broad region of a gene, in a single therapeutic, offer the potential of developing a single editing therapy able to treat multiple patients with different pathogenic variants. Recent reviews of promising genome editing approaches for the treatment of numerous genetic diseases include Anzalone et al. (2020), Porto et al. (2020), and Newby and Liu (2021).

## 4 Gene therapy for GSD-Ib

The development of clinical gene therapies for GSD-Ib, a deficiency in the G6P transporter (G6PT), is less well developed than those for GSD-Ia, because in addition to the metabolic phenotype of GSD-Ia, GSD-Ib patients also manifest neutropenia and myeloid dysfunction (Chou et al., 2002; Chou et al., 2010a; Chou et al., 2010b; Chou et al., 2015). Therefore, the current perspective is that successful gene therapy for GSD-Ib will require targeting not only the liver but also the hematopoietic stem and progenitor cells (HSPC). Advancements in HSPC gene therapy, including gene editing have been extensively reviewed by Ferrari et al. (2021).

In initial studies of liver-directed gene augmentation therapy for GSD-Ib, Yiu et al. (2009) infused newborn *G6pt*−/− mice with rAAV8-CBA-G6PT, an AAV2/8 vector expressing human G6PT directed by the CBA promoter/CMA enhancer, and showed that the treatment markedly prolonged their survival, providing a long-term metabolic correction alongside a transient myeloid correction. Hepatic G6PT activity was 50% of WT levels at 2 weeks post-infusion but then declined rapidly leveling off at 3% of WT levels from age 6–72 weeks. However, by ages 52–72 weeks, the five treated mice exhibited excessive hepatic glycogen storage and hepatic steatosis, and two mice (40%) developed HCA/HCC. This suggests that 3% of normal hepatic G6PT activity does not prevent HCA/HCC development and restoring an effective level of G6PT has a higher bar than G6PC.

To improve expression, the native G6PT promoter/enhancer was examined. The minimal *G6PT* promoter/enhancer (miGT) is contained within nucleotides −610 to −1 upstream of the G6PT translation start site (Hiraiwa and Chou, 2001). Kwon et al. (2017) first evaluated the efficacy of two constructs: rAAV8-miGT-G6PT, a double-stranded vector expressing human G6PT directed by miGT; and rAAV8-GT-G6PT, a single-stranded vector expressing human G6PT directed by the 1.62 kb human *G6PT* promoter/enhancer (GT). Again, the findings contrasted with those of G6PC. While the rAAV8-miGT-G6PT vector markedly prolonged the survival of the *G6pt*−/− mice, only10% of the rAAV8-GT-G6PT-treated *G6pt*−/− mice lived to age 12 weeks. The reason for the lower efficacy of the longer promoter over the minimal promoter has not been elucidated yet but might suggest there are DNA silencer and/or genetic suppressor element binding sites in the longer sequence.

Since the human *G6PC* promoter/enhancer (GPE) expresses well in liver, Kwon et al*.* (2017) constructed rAAV8-GPE-G6PT, a single-stranded G6PT-expressing vector directed by the 2.8-kb human GPE. They showed that both rAAV8-GPE-G6PT and rAAV8-miGT-G6PT directed persistent hepatic G6PT expression. The rAAV8-GPE-G6PT, driven by the extended *G6PC* promoter/enhancer, was 4-fold more efficient in directing hepatic *G6PT* activity, than the rAAV8-miGT-G6PT vector, driven by the *G6PT* minimal promoter/enhancer (Kwon et al*.,* 2017), consistent with the additional positive hepatic regulatory elements identified in the GSD-Ia studies.

The hepatic G6PT activity required to maintain glucose homeostasis and prevent tumor formation was then examined over a 78-week study (Kwon et al*.,* 2017). The *G6pt*−/− mice that expressed ≥6% of normal hepatic G6PT activity had a normal liver phenotype. They had no detectable anti-human G6PT antibodies, did not develop HCA, and did not develop the age-related obesity or insulin resistance. However, *G6pt*−/− mice that expressed <6% of normal hepatic G6PT activity had an increased risk of HCA, establishing the minimum activity required to prevent tumor formation. As with the GSD-Ia studies, full restoration of normal hepatic G6PT activity was not required for meaningful therapeutic benefits in liver-directed gene therapy for murine GSD-Ib (Kwon et al*.,* 2017). The preclinical studies have established gene therapy for GSD-Ib to be safe and efficacious in treating metabolic complications of GSD-Ib and may support development of a therapeutic for liver-directed gene therapy for human GSD-Ib patients.

One interesting finding in rAAV-mediated gene therapy for GSD-Ib is that there appears to be a functional feedback mechanism where a decrease in hepatic G6PT expression is offset by an increase in the expression of hepatic G6Pase-α (Kwon et al., 2017). This is reminiscent of the inverse relationship where an elevation in hepatic G6PT activity correlated with low levels of hepatic G6Pase-α activity (Kim et al., 2015; Kim et al., 2017b). Since other G6P transporters cannot couple with G6Pase-α (Pan et al., 2011), it is likely that the G6Pase-α/G6PT co-dependence is, at least in part, due to a direct physical interaction between the proteins in the ER membrane.

**FIGURE 10:**
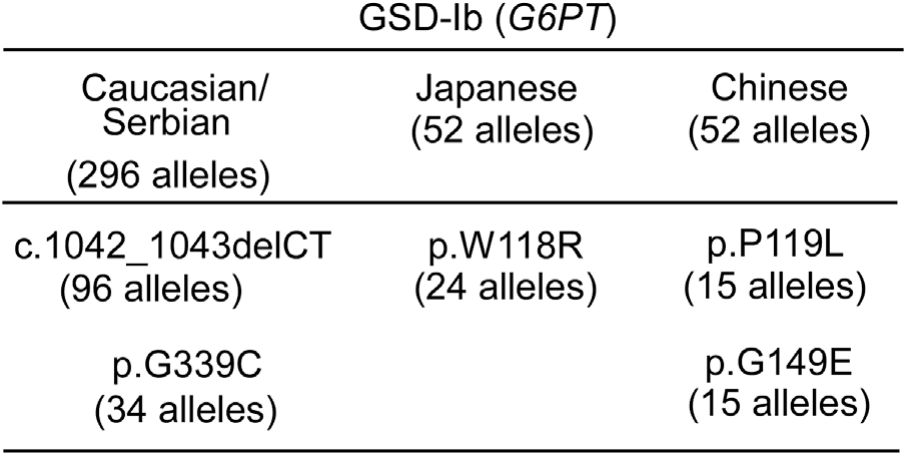
Ethnic differences in the prevalent G6PT variants identified in GSD-Ib patients (Fokkema et al., 2021) "https://databases.lovd.nl/shared/variants/SLC37A4?search_var_status=%3D%22Marked%22%7C%3D%22Public%22 " accessed in March 2023. The total number of *SLC37A4* (*G6PT*) alleles genotyped for each ethnicity (Chou et al., 2010a; Chou et al., 2010b; Chou et al., 2018) is given in the heading. Only Pathogenic variants with allele counts greater than 10 are listed.

While currently less developed, the same alternative gene therapeutic approaches outlined above for GSD-Ia may also be applied to GSD-Ib. Several prevalent *G6PT* pathogenic variants have been identified in GSD-Ib patients (Chou et al., 2018) (Figure 10) that may be addressed by base editing (Komor et al., 2016; Gaudelli et al., 2017; Nami et al., 2018; Ryu et al., 2018; Molla and Yang, 2019) or prime editing (Anzalone et al., 2020; Porto et al., 2020; Newby and Liu, 2021).

## 5 Concluding remarks and future perspectives

As a disease with neonatal onset, GSD-I is life threatening from birth. Genetic therapies may offer an opportunity to move away from the current strict dietary regimes that require constant monitoring, and are disruptive to daily life and sleep routines, particularly in the young. Gene augmentation therapy for GSD-Ia, now entering a phase III clinical trial, offers a single therapeutic administration. The window for single dose therapeutic intervention by gene augmentation in humans still needs to be defined. Preclinical data in mice suggest it may be most appropriate for patients with mature livers. The outcomes of the clinical trials NCT03517085, treating patients 18 years and older, and NCT05139316, treating patients 8 years and older may help clarify the therapeutic window. Transient approaches using multiple dosing, like the mRNA augmentation that has just entered a phase I/II clinical trial for GSD-Ia, may offer a valuable pediatric option, although less attractive for longer-term therapy.

Gene editing techniques close to clinical trial may offer a single administration with permanent, non-inheritable, liver cell genome correction, but currently each pathogenic variant requires a customized therapeutic, each independently validated in a clinical trial. While there is significant diversity in the pathogenic variants of the GSD-Ia population, there are populations that may benefit more from this variant-specific approach. For instance, in the Japanese and Korean GSD-Ia populations, a single pathogenic variant, c.648G>T accounts for over 85% of *G6PC* variants and in Caucasian/Jewish GSD-Ia population, two pathogenic variants p.R83C and p.Q347X account for ∼56% of *G6PC* variants (Figure 9). For successful gene augmentation, the durability of gene expression over decades of life is required, and if not life-long, a route to re-administration, avoiding adverse immune responses will be required. For gene editing, understanding the precision of editing, the minimum number of cells requiring correction for efficacy, the risk of off-target effects, and genomic rearrangements, along with the durability is important. Looking to the future, a gene editing technology that can account for all pathogenic variants by replacing a whole gene or placing an additional gene cassette in a safe harbor genomic locus may be attractive.
